# Risk following a severe acute respiratory coronavirus virus 2 (SARS-CoV-2) exposure from a nocturnal hemodialysis patient utilizing continuous positive airway pressure (CPAP)

**DOI:** 10.1017/ice.2020.291

**Published:** 2020-06-11

**Authors:** Christopher F. Lowe, Mercedeh Kiaii, Laila Aparicio, Leila Chinybaeva, Sandy Coughlin, Inna Sekirov, Muhammad G. Morshed, Victor Leung

**Affiliations:** 1 Infection Prevention and Control, Providence Health Care, Vancouver, Canada; 2 Department of Pathology and Laboratory Medicine, University of British Columbia, Vancouver, Canada; 3 Division of Nephrology, Providence Health Care, Vancouver, Canada; 4 Occupational Health and Safety, Providence Health Care, Vancouver, Canada; 5 BCCDC Public Health Laboratory, Vancouver, Canada

*To the Editor—*Hemodialysis units are challenging environments to implement infection prevention and control (IPAC) recommendations for coronavirus disease 2019 (COVID-19).^1^ The Centers for Diseases Control and Prevention (CDC) recommends airborne and contact precautions for patients with suspected or confirmed cases of COVID-19.^2^ In contrast, the Public Health Agency of Canada has recommended droplet and contact precautions, only recommending airborne precautions for aerosol-generating medical procedures (AGMPs).^3^ Both recommendations are difficult to implement in hemodialysis facilities, which are predominantly open, pod-based units with limited or no single rooms, but recommendations have been developed specifically for dialysis facilities to prevent severe acute respiratory coronavirus virus 2 (SARS-CoV-2) transmission.^4^ We describe our experience following an exposure to a nocturnal hemodialysis cohort as a result of an AGMP in a patient subsequently confirmed to have coronavirus disease 2019 (COVID-19).

## Methods

The study was conducted at a tertiary-care hospital in Vancouver, Canada. Nocturnal hemodialysis patients are routinely dialyzed in the same pod, which accommodates 12 patients.

Laboratory testing for SARS-CoV-2 was conducted on the cobas 6800 (Roche Molecular Diagnostics, Pleasanton, CA), targeting the Orf-1a and envelope (E) genes. COVID-19 point-of-care serology testing (COVID-19 IgM/IgG antibody test, Artron, Burnaby, Canada) was performed at the provincial reference laboratory.

According to institutional infection prevention and control policies, patients with symptoms consistent with COVID-19 are tested for SARS-CoV-2 with a nasopharyngeal swab. In our hemodialysis unit, patients’ temperatures are assessed on arrival and discharge, and any patients with a temperature of ≥37°C are tested. Suspected or confirmed cases are placed on droplet and contact precautions in a separate pod away from the main cohort. For all hemodialysis patients, staff utilize gowns, gloves, procedure masks, and eye protection during the initiation and conclusion of the hemodialysis procedure. Our institution also introduced a policy of universal surgical mask and eye protection for healthcare workers (HCWs) in clinical areas. Contact tracing included staff or patients exposed to the index patient up to 48 hours before symptom onset.

The Research Ethics Board of the University of British Columbia/Providence Health Care Research Institute approved this study.

## Results

### Index case

After completing a nocturnal hemodialysis run, the patient had an oral temperature of 37.3°C. He endorsed general malaise and decreased appetite 6 days prior to the current session. He denied any respiratory or gastrointestinal symptoms, and did not have any sick contacts. Unbeknownst to the staff, he used his continuous positive airway pressure (CPAP) machine during nocturnal dialysis. A nasopharyngeal swab was collected and positive for SARS-CoV-2 (Orf-1a cycle threshold (Ct) = 18.8; E Ct = 19.14). Subsequently, the patient was rescheduled to daytime dialysis and was advised not to use CPAP during hemodialysis. He was isolated from other patients and dialyzed in a separate pod on contact/droplet precautions until he had 2 nasopharyngeal swabs negative for SARS-CoV-2 separated by 24 hours. In total, 4 nocturnal sessions (~8 hours per session) occurred in which this patient was considered infectious based on symptom onset.

### Exposed patients

There were 11 patients in the same nocturnal cohort. All were monitored for 14 days after exposure, including routine symptom monitoring prior to each hemodialysis run, and they were advised to self-isolate at home. None of the 11 exposed patients developed any symptoms, and all were negative for SARS-CoV-2 from nasopharyngeal swabs on day 5 and 14 after exposure. Serology was tested on day 19 and day 33 after exposure. Overall, 10 patients were negative for IgG and IgM; 1 patient had a faint IgM band at day 19, but the IgM/IgG antibody test was negative at day 33.

### Exposed HCW

Overall, 10 nurses and 2 renal technologists were exposed. All HCWs adhered to the universal procedure mask and eye protection policy. The HCWs were asked to self-isolate at home and to get tested if they developed any symptoms consistent with COVID-19. During the 14 days after exposure, 3 staff reported COVID-19 symptoms. Two visited an HCW screening site, and their nasopharyngeal swabs were negative for SARS-CoV-2. The third HCW reported self-limited nausea/vomiting postexposure day 3 but did not subsequently get tested.

## Discussion

We report follow-up of 11 patients and 12 HCWs exposed to SARS-CoV-2, in which an AGMP occurred without airborne/contact precautions in nocturnal hemodialysis. This exposure occurred over 4 sessions, with ~32 hours of exposure time. Defining the level of exposure can depend on numerous factors including appropriateness of personal protective equipment, contact with bodily fluids, duration of exposure, and presence of an AGMP. In our case, although the CDC defines this exposure as medium risk for staff,^5^ the prolonged duration of exposure to an ongoing AGMP represents higher risk. For the patients, who were not wearing procedure masks, the exposure would have been considered high risk. Reassuringly, none of the patients at highest risk developed COVID-19 symptoms, and they were negative by polymerase chain reaction assay (PCR) and serology. Although 1 patient developed a faint IgM band, repeat testing at day 33 was negative suggesting an initial false positive. Follow-up of patients and HCWs was similar to an incident reported in critical care in which SARS-CoV-2 was diagnosed on extubation. In this setting, none of the 35 HCWs exposed to an AGMP for at least 10 minutes developed COVID-19 symptoms or tested positive for SARS-CoV-2.^6^

In clinical practice, situations arise such as this case where delayed diagnosis of COVID-19 contributed to the exposure of 23 patients and staff. Contact tracing did not suggest transmission, and transmission was likely mitigated by existing IPAC precautions on the unit such as hand hygiene, universal procedure mask and eye protection in clinical areas and droplet and contact precautions for the start and end of each hemodialysis procedure.
